# LARP1 knockdown inhibits cultured gastric carcinoma cell cycle progression and metastatic behavior

**DOI:** 10.1515/biol-2022-0806

**Published:** 2024-01-22

**Authors:** Xin Liu, Wei-Ming Zhang, Nuo Meng, Lian-Jie Lin, Guo-Du Tang

**Affiliations:** Department of Gastroenterology, The First Affiliated Hospital of Guangxi Medical University, No. 6, Shuangyong Road, Nanning, Guangxi 530021, P. R. China; Department of Gastroenterology, Wuming Hospital Affiliated to Guangxi Medical University, Nanning, Guangxi, 530199, P. R. China; Department of Radiotherapy, Wuming Hospital Affiliated to Guangxi Medical University, Nanning, Guangxi, 530199, P. R. China

**Keywords:** LARP1, gastric carcinoma cells, apoptosis, invasion, migration

## Abstract

This study aimed to clarify the role of la-related protein 1 (LARP1) in cell cycle progression and metastatic behavior of cultured gastric carcinoma (GC) cells. To do that, LARP1 expression was detected in clinical GC tissues and cell lines using quantitative real-time polymerase chain reaction (qRT-PCR) and western blotting. The cell viability, apoptosis, cell cycle, migration, invasion, and cell growth were examined using a Cell Counting Kit-8, Annexin V-FITC staining, propidium iodide staining, Transwell migration and invasion assays, and colony formation assays after LARP1 knockdown. Phosphatidyl inositol 3-kinase (PI3K) and AKT1 mRNA and protein expression levels of PI3K, p-AKT1, AKT1, p-BAD, p-mTOR, and p21 in si-LARP1 transfected GC cells were determined using qRT-PCR and western blotting. Here, we've shown that LARP1 expression was upregulated in human GC tissues and KATO III cells. LARP1 knockdown inhibited GC cell proliferation, cell cycle progression, migration, invasion, and colony formation and promoted apoptosis. In si-LARP1-transfected KATO III cells, the mRNA expression levels of PI3K and AKT1, PI3K protein expression, and the p-AKT1/AKT1 ratio were significantly suppressed. p-mTOR and p-BAD were significantly decreased, whereas p21 was significantly increased in si-LARP1-transfected KATO III cells. In conclusion LARP1 knockdown induces apoptosis and inhibits cell cycle progression and metastatic behavior via PI3K/AKT1 signaling in GC cells.

## 1 Introduction

Gastric carcinoma (GC) is one of the five most common cancers worldwide and is a major contributor to cancer-related deaths [1]. Despite the introduction of new diagnostic and therapeutic strategies, the prognosis remains poor, and the mortality rate is high [2]. Widely explored chemotherapeutic agents have reached a plateau, where the median overall survival is no more than 1 year [3], and the main research direction is to understand the molecular biology of tumorigenesis. Understanding the cell signaling mechanisms that promote carcinogenesis can offer valuable information for the development of new and efficient therapies [4].

As a multifactorial disease, cancer can be influenced by both environmental and genetic factors, and the multiple stages of carcinogenesis are controlled by the gradual development of mutations in gene expression and epigenetic changes. These genetic alterations lead to cancer progression by affecting the fate of cancer cells (e.g., proliferation, apoptosis, and metastasis) [5]. With the recent advancements in understanding the complex genetic changes underlying human cancers, identifying molecularly targeted therapies that will benefit patients is significant [6]. However, the mechanisms underlying GC cell survival, invasion, and metastasis are not fully understood.

As a highly evolutionarily conserved RNA-binding protein, la-related protein 1 (LARP1) is well known as a member of the LARP family (including LARP1, LARP3, and LARP7), each of which carries a conserved RNA-binding La domain and an RNA recognition mode-like domain [7]. Functionally, LARP1 primarily plays the role of post-transcriptional regulator of genes by recognizing the 5′ terminal oligopyrimidine (5′ TOP) motif characterization or 3′ untranslated region of mRNA [8,9]. Abnormal levels and functions of LARP1 are associated with disease progression [10]. Changes in LARP1 levels are associated with cancer progression. Upregulated LARP1 expression in hepatocellular carcinoma and lung carcinoma is correlated with poor prognosis [11]. Increased levels of LARP1 in cervical cancer facilitate the migration and invasion of cancer cells by acting on mRNAs rich in carcinogenic transcripts [12]. LARP1 is essential for the survival of epithelial ovarian cancer cells and promotes ovarian cancer tension and chemotherapy resistance [13]. These studies indicate that LARP1 acts as an oncogene and is involved in carcinoma progression. To date, limited evidence has demonstrated the role of LARP1 in GC.

This study determined the expression pattern of LARP1 in clinical GC tissues and investigated the LARP1 function in GC cell survival and metastasis *in vitro*. The discovery of new therapeutic targets is expected to improve the therapeutic effects in GC.

## 2 Materials and methods

### 2.1 Clinical GC tissues

Six patients (two females and four males) with GC at the antrum of the stomach were hospitalized between January 2020 and August 2020 and underwent resection. Paired GC and para-carcinoma tissues were obtained after obtaining informed consent prior to sample collection. A part of the tissue was used for pathological examination and a part was used for quantitative real-time polymerase chain reaction (qRT-PCR) detection. Tissues for qRT-PCR detection were placed in liquid nitrogen immediately after excision and stored at −80°C before analysis. Pathological results showed that the samples were in TNM stages I and II. Sample acquisition and experimental procedures were conducted in accordance with the Ethics Committee of Wuming Hospital Affiliated to Guangxi Medical University.


**Informed consent:** Informed consent has been obtained from all individuals included in this study.
**Ethical approval:** The research related to human use has been complied with all the relevant national regulations, institutional policies and in accordance with the tenets of the Helsinki Declaration, and has been approved by the Ethical Committee of Wuming Hospital Affiliated to Guanxi Medical University.

### 2.2 Cell lines

A normal human gastric mucosa cell line (GES-1) and three human GC cell lines (SNU-1, NCI-N87, and KATO III) were purchased from the Cell Bank of the Chinese Academy of Sciences (Shanghai, China). All cells were maintained in 37°C and 5% CO_2_ cell incubator using RPMI-1640 medium (11875101, Thermo Fisher Scientific, Waltham, MA, USA), supplemented with 10% fetal bovine serum (10091148, Thermo Fisher Scientific) and 1% penicillin–streptomycin (15140122, Thermo Fisher Scientific).

### 2.3 qRT-PCR

Before RNA isolation, tissue samples were homogenized, and cells with various treatments were collected by washing with phosphate-buffered saline (PBS) (B548117-0500, Sangon Biotech, Shanghai, China). RNAiso Plus (TaKaRa, Kyoto, Japan) was used to lyse the cells, and the chloroform isoamyl alcohol method was used to extract total RNA from the lysate. The purity and concentration of RNA were determined using a microplate reader (Thermo Fisher Scientific). For reverse transcription, RNA was mixed with primeScript RT Master MIX (RR036A, TaKaRa), and a reaction procedure was performed at 37°C for 60 min and at 85°C for 5 s. The cDNA product was amplified using Power SYBR Green PCR Master Mix (4367659, Thermo Fisher Scientific). The primers used are listed in Table 1. Relative mRNA expression was calculated using the 2^‒ΔΔCT^ method with GAPDH as an internal reference.

**Table 1:** Primers used in qRT-PCR

Gene	Direction	Sequence (5′−3′)
LARP1	Forward	ACACAAGTGGGTTCCATTACAAA
	Reverse	CTCCGCGATTGGCAGGTAT
PI3K	Forward	CCACGACCATCATCAGGTGAA
	Reverse	CCTCACGGAGGCATTCTAAAGT
AKT1	Forward	AGCGACGTGGCTATTGTGAAG
	Reverse	GCCATCATTCTTGAGGAGGAAGT
GAPDH	Forward	TGACAACTTTGGTATCGTGGAAGG
	Reverse	AGGCAGGGATGATGTTCTGGAGAG

### 2.4 siRNA transfection

Three LARP1 siRNAs were designed and synthesized by BioTen Co., Ltd. (Shanghai, China). Before transfection, approximately 6 × 10^5^/well of GC cells were seeded in a six-well plate and kept at 37°C and 5% CO_2_ for 24 h. LARP1 siRNA mix with Lipofectamine 2000 (11668027, Thermo Fisher Scientific) was added to each well and incubated for 6 h. After removing the siRNA mixture solution, the cell culture was continued in a complete medium. After 48 h of transfection, the cells were harvested to determine the effect of LARP1 siRNA.

### 2.5 Cell viability assay

A total of 5 × 10^3^/well of GC cells were seeded in a 96-well plate and incubated at 37°C and 5% CO_2_ for 24 h. Cells were harvested after 24, 48, and 72 h of transfection. Cell viability was detected using a cell counting kit-8 (C0037, Beyotime) based on the protocols recommended by the manufacturer.

### 2.6 Apoptosis detection

Cultured cells were digested using trypsin and collected after centrifugation at 1,000 × *g* for 5 min. Apoptosis was determined using the FITC Annexin V Apoptosis Detection Kit (556420; BD Biosciences). In brief, after washing with PBS, cells were resuspended with 195 μL of Annexin V-FITC binding buffer. Cells were then added with 5 μL of Annexin V-FITC and 10 μL of propidium iodide (PI) staining solution. Incubation staining was performed for 20 min at room temperature (20–25°C) away from light. The cells were subjected to apoptosis detection using a FACSCalibur flow cytometer (BD Biosciences).

### 2.7 Cell cycle distribution detection

The cultured cells were resuspended in PBS and mixed with 4 mL of 70% ethanol (−20°C precooled). Cells were fixed overnight in a 4°C refrigerator. After washing with PBS, the fixed cells were resuspended in PBS supplemented with 50 μg/mL RNase A and kept in a 37°C water bath for 30 min. PI at a final concentration of 50 μg/mL (ST512, Beyotime) was added to stain the cells for 30 min away from light. The cells were subjected to cell cycle distribution analysis using a FACSCalibur flow cytometer (BD Biosciences, USA).

### 2.8 Transwell assay

Cultured cells were collected after trypsin digestion and resuspended in 5 mL of sterile PBS for cell counting. The cells were maintained in a serum-free medium to adjust the cell density to 5 × 10^5^/mL. The upper transwell was supplemented with 200 μL of cell suspension and cultured for 48 h. The migrated and invaded cells were fixed in 4% paraformaldehyde for 20 min. Staining was performed using a crystal violet staining solution (C0121, Beyotime). For the Transwell invasion assay, the transwell chamber was pre-coated with Matrigel (354234, Corning, MA, USA).

### 2.9 Colony formation

After 24 h of transfection, the cells were digested with trypsin and resuspended. The cell suspension was seeded in a six-well plate at 200 cells/well and cultured for 14 days. Colonies on the plates were fixed with 4% paraformaldehyde (80096618; Sinopharm, Shanghai, China) for 4 min. Staining was performed using a crystal violet staining solution (C0121, Beyotime). After the unstained cells were washed away, the plates were photographed, and colonies (containing >50 cells) were counted.

### 2.10 Western blot

Radio immunoprecipitation assay lysis (P0013B, Beyotime) buffer containing 1 mM phenylmethanesulfonylfluoride (ST506, Beyotime) was added to GC cells and centrifuged at 12,000 × *g* and 4°C for 10 min. Proteins were quantified using a bicinchoninic acid kit (PL212989, Thermo Fisher Scientific). After adding the loading buffer, the proteins were separated via sodium dodecyl sulfate-polyacrylamide gel electrophoresis and transferred onto a polyvinylidene fluoride (PVDF) membrane (IPVH00010, Millipore, Boston, MA, USA). Subsequently, the PVDF membrane was blocked by 5% defatted milk for 1 h at 37°C and incubated with primary antibodies of LARP1 (ab86359; Abcam, Cambridge, UK; 1:2,000), phosphatidyl inositol 3-kinase (PI3K) (20584-1-AP; Proteintech, Wuhan, Hubei, China; 1:500), p-AKT (28731-1-AP; Proteintech; 1:1,000), AKT1 (60203-2-Ig; Proteintech; 1:5,000), p-BAD (5284T; CST, Boston, MA, USA; 1:1,000), p-mTOR (5536T; CST; 1:1,000), and GAPDH (10494-1-AP; Proteintech; 1:5,000) overnight at 4°C. On the second day, (H + L)-HRP secondary antibody (115-035-003; Jackson ImmunoResearch, West Grove, PA, USA; 1:5,000) was added and incubated at 37°C for 2 h. Protein expression was determined using an ECL system (Millipore) and quantified using ImageJ software.

### 2.11 Statistical analysis

GraphPad Prism 7.00 version was used for statistics and analysis of data. An unpaired *t*-test was used to analyze differences between two groups, and a one-way analysis of variance following Tukey’s multiple comparison test was used for analyzing differences among multiple groups. *P* values <0.05 were considered statistically significant.

## 3 Results

### 3.1 LARP1 expression was upregulated in human GC tissues and GC cell lines

**Figure 1:**
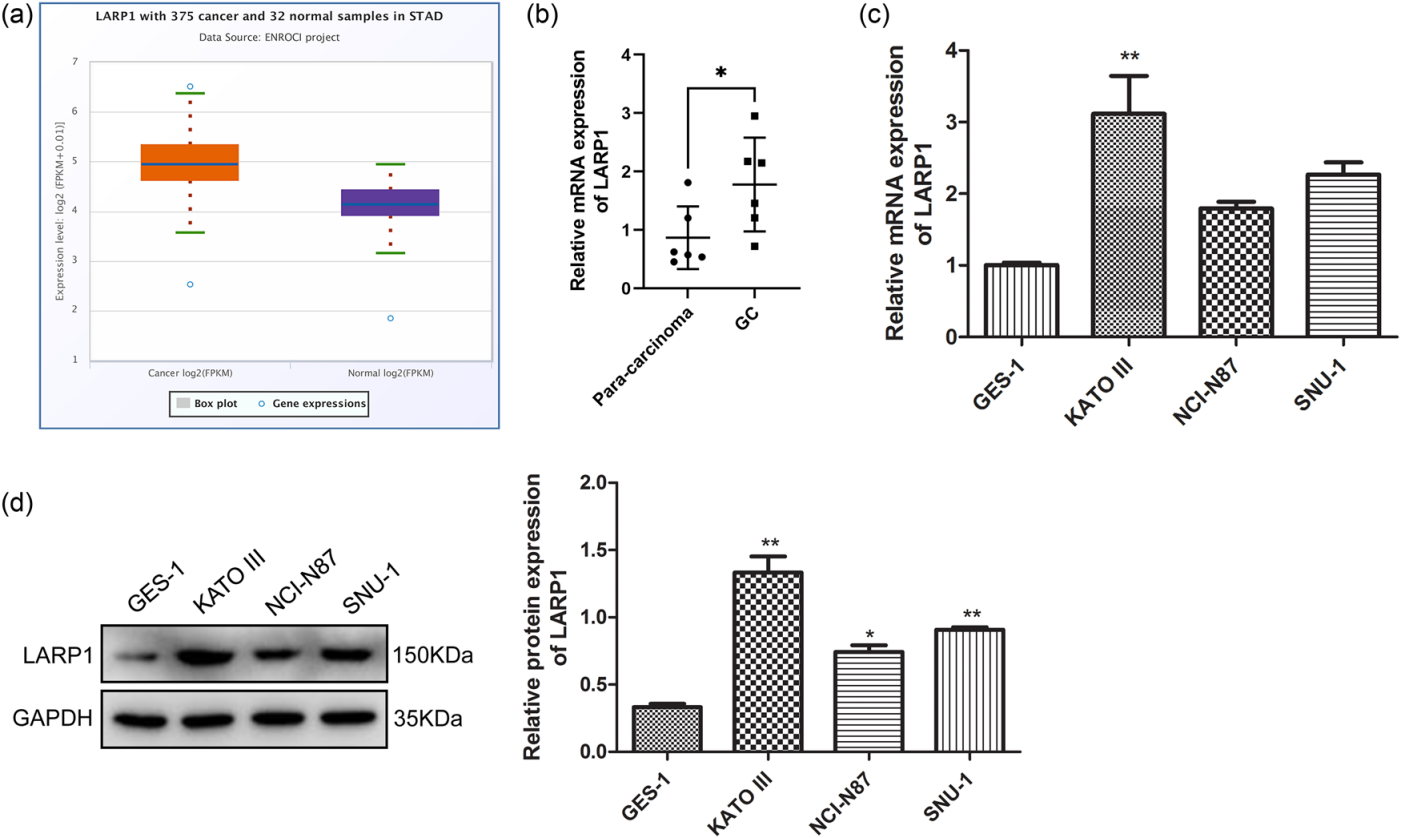
LARP1 expression level in clinical GC tissues and GC cell lines. (a) mRNA expression levels of LAPR1 in stomach cancer (STAD) and normal samples were searched in the ENCORI platform. (b) LARP1 level was compared between six pairs of GC tissues and para-carcinoma tissues using qRT-PCR. (c) The mRNA expression of LARP1 was compared between a normal gastric mucosa cell line (GES-1) and three GC cell lines (KATO III, NCI-N87, and SNU-1). (d) The protein expression of LARP1 was compared between GES-1 and three GC cell lines (KATO III, NCI-N87, and SNU-1) using a western blot. *P < 0.05, **P < 0.01 compared with para-carcinoma tissue or GES-1.

To determine the expression pattern of LARP1 in GC, we first compared its expression in GC and normal tissues using the ENCORI (Starbase, https://rnasysu.com/encori/index.php). As shown in Figure 1a, the expression level of LARP1 was significantly higher in stomach cancer samples than in normal samples (*P* = 5.6 × 10^−20^). To further verify the results, we measured its levels in six pairs of GC and para-carcinoma tissue samples in our cohort samples. qRT-PCR analysis showed that LARP1 mRNA was expressed at higher levels in GC tissues than in paracarcinoma tissues (Figure 1b). The LARP expression pattern was further verified in three GC cell lines. As shown in Figure 1c, compared to the normal gastric mucosa cell line (GES-1), LARP1 mRNA levels were significantly increased in KATO III cells (*P* < 0.01) but not in NCI-N87 and SNU-1 cells (*P* > 0.05). The protein expression of LARP1 was significantly higher in the three GC cell lines than in the normal gastric mucosal cell line (Figure 1d, *P* < 0.05). Therefore, the KATO-III cells were selected for subsequent experiments.

### 3.2 LARP1 knockdown inhibited GC cell survival and promoted cell apoptosis

**Figure 2:**
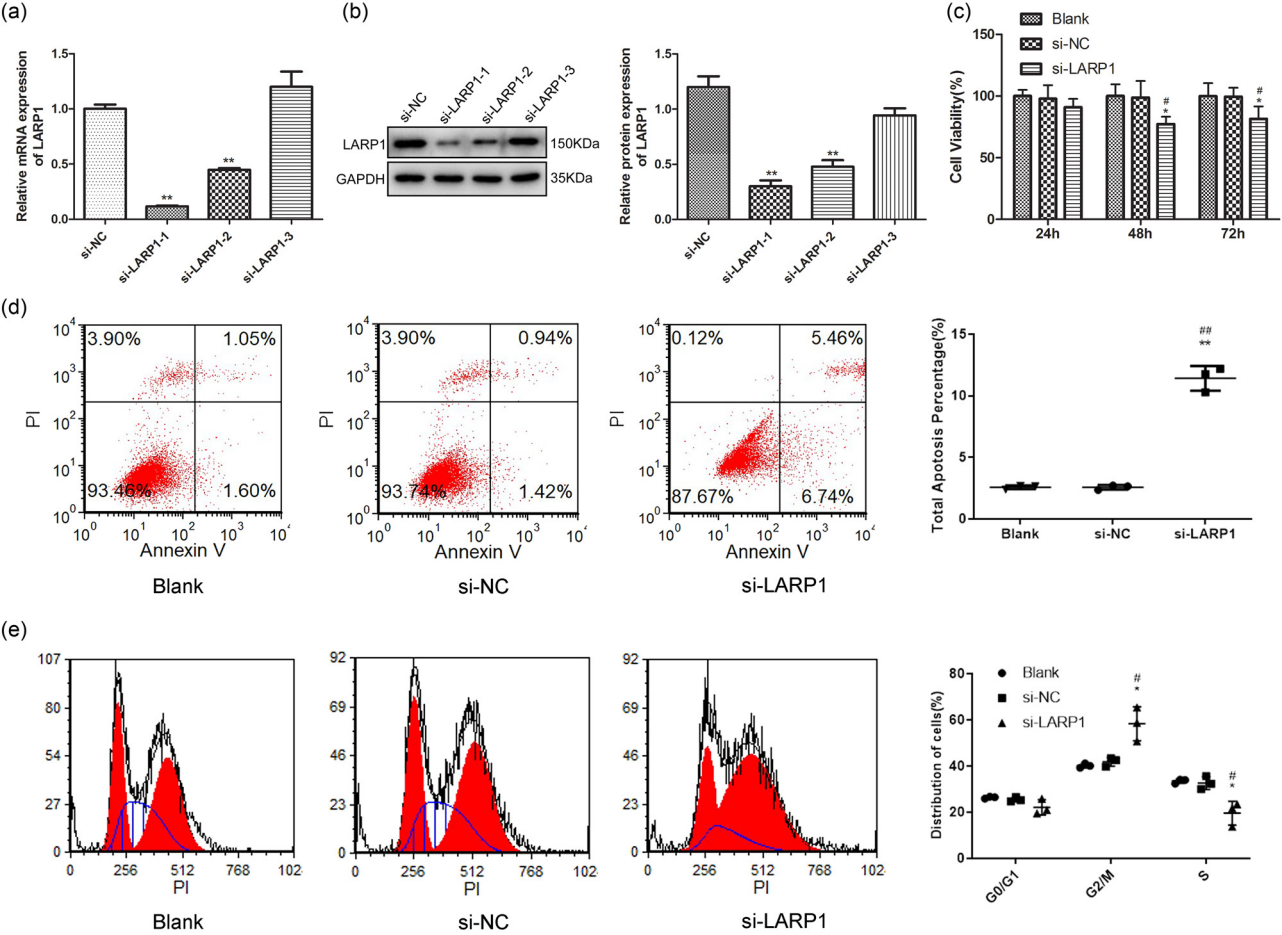
Effect of LARP1 knockdown on GC cell viability, apoptosis, and cell cycle distribution. (a) LARP1 mRNA expression was determined in KATO III cells transfected with three RNA interfering sequences. (b) LARP1 protein expression was determined in KATO III cells transfected with three RNA interfering sequences. (c) Cell viability was analyzed in KATO III cells 24, 48, and 72 h after si- LARP1 transfection. (d) Cell apoptosis was determined using Annexin V-FITC flow cytometric analysis in KATO III cells after 48 h of si-LARP1 transfection. (e) Cell cycle distribution was determined using propidium iodide (PI) flow cytometric analysis in KATO III cells after 48 h of si-LARP1 transfection. *P < 0.05, **P < 0.01 compared with the blank group. ^#^ *P* < 0.05, ^##^ *P* < 0.01 compared with the si-NC group.

Given the high levels of LARP1 in GC, the next step was to establish a knockdown LARP1 cell line and verify the function of LARP1 in GC cell survival. Three RNA-interfering sequences for LARP1 (si-LARP1-1, si-LARP1-2, and si-LARP1-3) were used to transfect KATO III cells, and si-LARP1-1 and si-LARP1-2 were effective in knocking down LARP1 expression (Figure 2a and b). si-LARP1-1, with the greatest interfering effect, was selected for subsequent experiments. In KATO III cells, at 48 and 72 h after si-LARP1 transfection, cell viability was remarkably decreased (Figure 2c), suggesting that LARP1 knockdown had an inhibitory effect on cell viability. Forty-eight hours of si-LARP1 transfection were used in subsequent experiments. Further experiments explored the cell apoptosis of si-LARP1-transfected KATO III cells. As shown in Figure 2d, compared with si-NC, LARP1 knockdown immensely increased the percentage of apoptotic cells. Cell cycle distribution analysis showed that cells with LARP1 knockdown had an increased G2/M phase fraction and a decreased S phase fraction, suggesting that the cell cycle was arrested in the G2/M phase fraction (Figure 2e). Overall, LARP1 knockdown inhibits GC cell survival and promotes cell apoptosis.

### 3.3 LARP1 knockdown restrained GC cell metastasis

**Figure 3:**
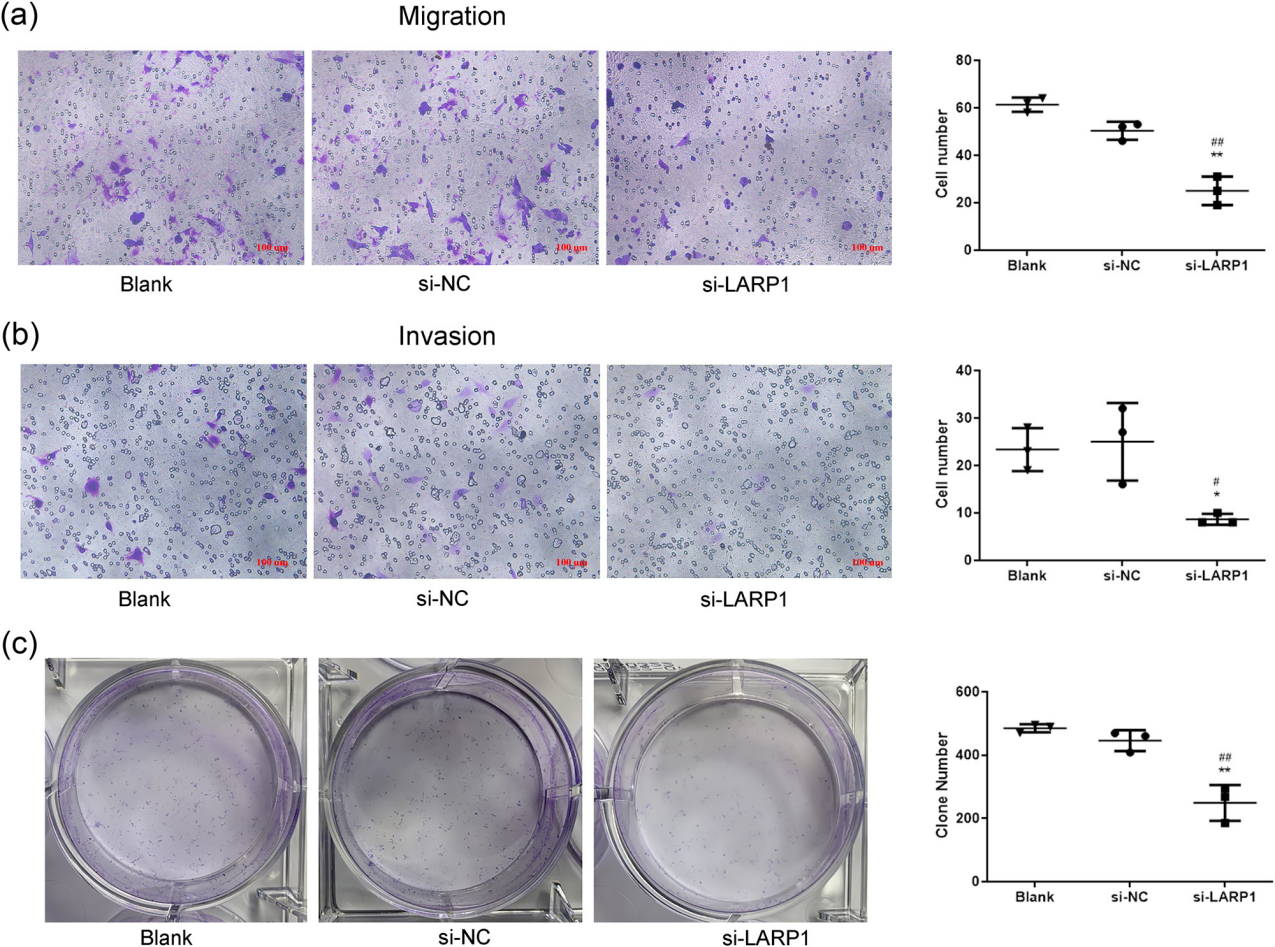
LARP1 knockdown inhibits GC cell migration, invasion, and colony formation. Transwell migration assay (a) and transwell invasion assay (b) were used to detect cell metastasis ability in si-LARP1-transfected KATO III cells. (c) Colony formation assay was used to evaluate cell growth in si-LARP1-transfected KATO III cells. *P < 0.05, **P < 0.01 compared with the blank group. ^#^ *P* < 0.05, ^##^ *P* < 0.01 compared with the si-NC group.

To further investigate the function of LARP1 in GC cell metastasis, cell invasion, migration, and colony formation abilities were determined in KATO III cells transfected with si-LARP1. Figure 3a showed that compared with si-NC, LARP1 knockdown markedly reduced the number of migrating cells. In the transwell invasion analysis, compared with si-NC, LARP1 knockdown reduced the number of invaded cells (Figure 3b). Regarding colony formation, KATO III cells with LARP1 knockdown had fewer colonies (Figure 3c). These data indicate that LARP1 knockdown restrained the metastasis and growth of GC cells.

### 3.4 LARP1 knockdown suppressed the PI3K/AKT1 signaling pathway

**Figure 4:**
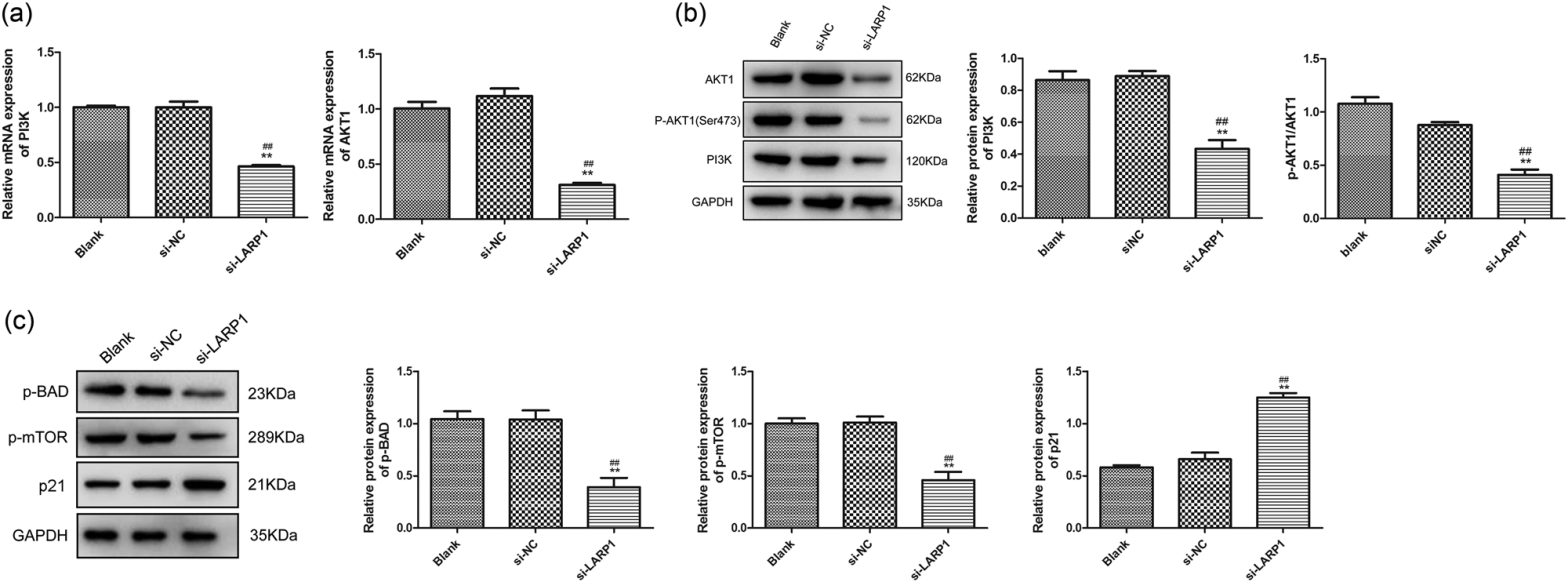
LARP1 knockdown suppresses the PI3K/AKT1 signaling pathway. (a) PI3K and AKT1 mRNA expression levels were determined using qRT-PCR. (b) Protein expression levels of PI3K, p-AKT1, and AKT1 were determined via western blotting. (c) Protein expression levels of p-BAD, p-mTOR, and p21 were determined via western blotting. **P < 0.01 compared with the blank group. ^##^ *P* < 0.01 compared with the si-NC group.

The PI3K/AKT1 signaling pathway is known to be involved in GC progression [14], and its inhibition promotes apoptosis and inhibits the metastasis of GC cells [15,16]. Next, we investigated whether the PI3K/AKT1 signaling pathway is the downstream target of LARP1 knockdown in KATO III cells. In si-LARP1 transfected KATO III cells, PI3K and AKT1 mRNA expression were significantly suppressed (Figure 4a). In addition, western blotting results suggested that PI3K and p-AKT1/AKT1 expression was significantly decreased in si-LARP1-transfected KATO III cells (*P* < 0.01). Given that LARP is involved in mTOR signaling, the downregulation of PI3K/AKT1 alone may not be equal to a reduction in cell viability. We further detected the expression of the PI3K/AKT downstream effectors involved in cell survival and proliferation, including p-mTOR, p-BAD, and p21. As shown in Figure 4c, p-mTOR and p-BAD were significantly decreased, whereas p21 was significantly increased in si-LARP1-transfected KATO III cells (*P* < 0.01).

## 4 Discussion

GC is a highly aggressive malignant tumor that constitutes a global health concern, and understanding its molecular characteristics will help identify more effective control strategies [17]. The current study elucidated the role of the RNA-binding protein LARP1 in GC cell cycle progression and metastatic behavior using *in vitro* cell experiments and demonstrated that LARP1 knockdown promoted cell apoptosis, arrested the cell cycle at the G2/M phase, and suppressed cell viability, migration, invasion, and colony formation in GC cells.

This study found that LARP1 was highly expressed in GC cells, and silencing of LARP1 inhibited GC cell viability and proliferation and promoted cell apoptosis and G2/M phase arrest. These results suggested that LARP1 plays an important role in regulating the malignant activity of cultured GC cells. LARP1 is an RNA-binding protein that can bind to mRNA with a 5′ TOP and regulate its stability and translation efficiency [18]. 5′ TOP mRNA mainly encodes proteins involved in translation and ribosome biogenesis. Therefore, LARP1 may affect protein synthesis and growth of GC cells by influencing the expression of 5′ TOP mRNA.

Additionally, LARP1 interacts with the mTORC1 complex in response to nutrient and growth factor signals. mTORC1 is a key regulator of cell growth, whose activity is regulated by LARP1 in pancreatic β-cells and hepatocytes [12]. Therefore, LARP1 may regulate the metabolism and proliferation of GC cells by participating in the mTORC1 signaling pathway. Moreover, this study found that silencing LARP1 caused G2/M phase arrest in GC cells, which may be related to the effect of LARP1 on cell cycle regulatory proteins. Some studies have shown that G2/M phase arrest is a mechanism of cell death induced by antitumor drugs that can trigger apoptosis or mitotic catastrophe [19–21]. Therefore, LARP1 may affect the fate of GC cells by regulating G2/M phase transition. However, how LARP1 senses and responds to cell cycle signals and how LARP1 interacts with other cell cycle regulators remain unclear, and further experiments are needed to investigate these issues.

As an oncogenic RNA-binding protein, LARP1 alters gene function and drives cancer development through abnormal changes in post-transcriptional regulation. Some studies have reported that metastasis, a crucial event in cancer progression, is affected by LARP1 expression. For example, Burrows et al. found that LARP1 regulates cell migration by facilitating the synthesis of proteins [22]. Mura et al. showed that LARP1 promotes cell growth, metastasis, and *in vivo* tumorigenesis in cervical cancer [12]. In the present study, GC cell invasion and migration were suppressed by LARP1 knockdown, indicating that LARP1 facilitates the motor ability of GC cells to invade and metastasize. LARP1 knockdown also inhibits colony formation in GC cells, suggesting that LARP1 plays an active role in proliferation and tumorigenesis. Therefore, we believe that LARP1 is critical for the aggressiveness of GC. However, the specific mechanisms require further investigation in animal studies and clinical trials.

Oxidative stress can lead to chronic inflammation, which, in turn, can cause multiple diseases, including cancer [23]. Natural products and their derived biomolecules that inhibit oxidative stress and inflammation are potential agents against cancer progression [24–27]. Inhibition of the PI3K/AKT signaling pathway was found to be effective in inhibiting angiogenesis, which plays a key role in cancer progression [28]. The present study revealed that PI3K and AKT1 expression was inhibited by the knockdown of LARP1. Activation of the PI3K and AKT1 cascades in many types of human cancers, including GC, drives cancer cell growth and survival, leading to tumor invasiveness and drug resistance [29,30]. The activation of PI3K and AKT1 and their roles in GC progression have been well described, including the promotion of survival and metastasis [31,32]. Activation of the PI3K/AKT1 pathway is involved in inhibiting the GC development of some anti-cancer substances, including drugs, functional proteins, and non-coding RNAs [33,34]. Although PI3K/AKT is a promising therapeutic target, AKT’s complex cellular functions of AKT and the activation of potential feedback signals limit AKT’s potency of AKT as a single drug [35]. A strategy targeting AKT in tumors with alterations in the PI3K pathway may benefit patients [36]. Our data showed that LARP1 knockdown suppressed the expression of PI3K and AKT1. Notably, there is limited evidence regarding the effect of LARP1 on the expression of PI3K and AKT1, and the specific regulatory mechanisms require further study.

Akt regulates cell growth by acting on the TSC1/TSC2 complex and mTORC signal transduction [37,38]. Akt affects cell proliferation by phosphorylating CDK inhibitors p21 and p27 [38]. Akt is a major regulator of cell survival and is regulated either directly by inhibiting pro-apoptotic proteins (such as BAD) or by inhibiting the production of pro-apoptotic signals via transcription factors [39]. Given that LARP is involved in mTOR signaling, the downregulation of PI3K/AKT1 alone may not be equal to a reduction in cell viability. We further detected the expression of the PI3K/AKT downstream effectors involved in cell survival and proliferation, including p-mTOR, p-BAD, and p21. The results indicated that p-mTOR and p-BAD were significantly decreased, whereas p21 was significantly increased in si-LARP1-transfected KATO III cells.

This study had some limitations. First, this study is only a preliminary investigation of the molecular mechanism of LARP1 in GC. The mechanistic analyses of the pathways involved in LARP1 expression are limited. Second, *in vivo* evidence is needed to confirm the role of LARP1 in the inhibition of metastatic behavior. Third, due to restrictions on funds and tissues, some experiments were lacking, such as repeated experiments in other cell lines or with another siRNA and immunohistochemical analysis of LARP1 in our cohort tissues.

## 5 Conclusion

**Figure 5:**
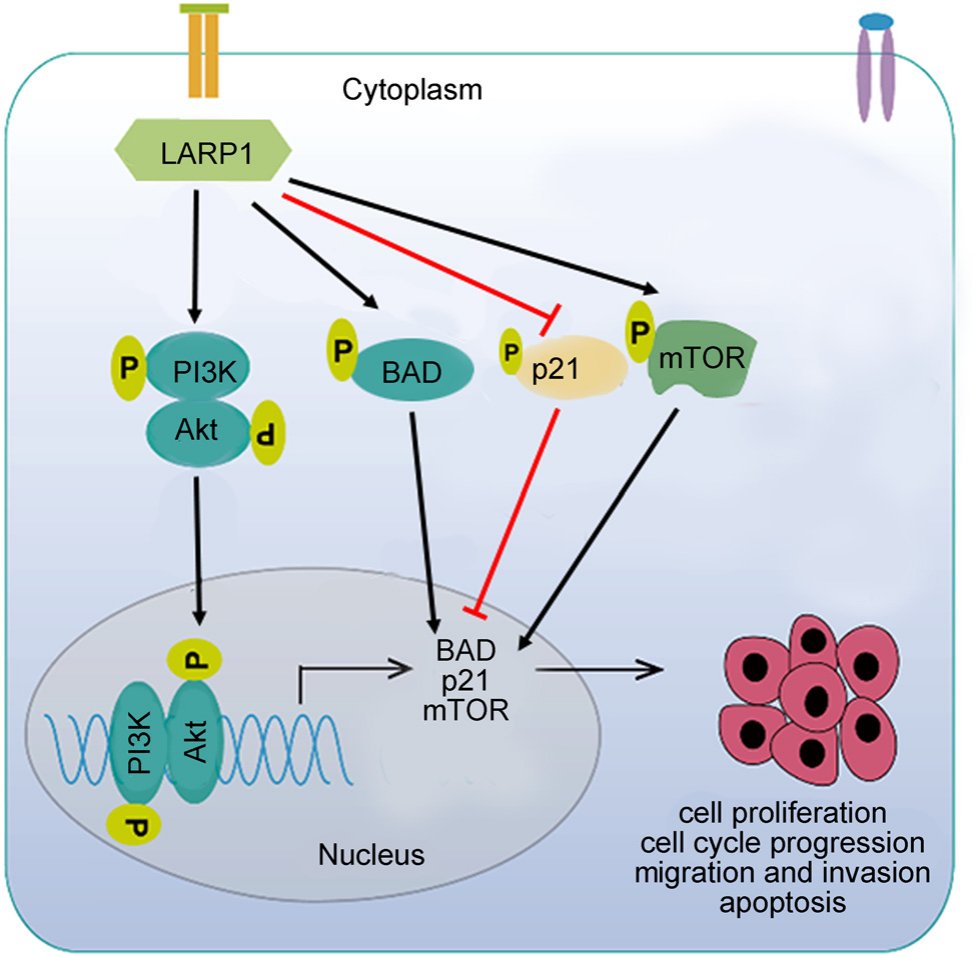
Molecular mechanism underlying the regulation of GC cell cycle progression and metastatic behavior by LARP1.

Our findings indicate that LARP1 is upregulated in GC and that LARP1 knockdown induces apoptosis and inhibits cell cycle progression and metastatic behavior in cultured GC cells, mediated by the PI3K/AKT pathway (Figure 5). Thus, LARP1 is a potential therapeutic target for the treatment of GC.
